# Attention deficit/hyperactivity disorder as an associated feature in OCTN2 deficiency with novel deletion (p.T440‐Y449)

**DOI:** 10.1002/ccr3.1316

**Published:** 2018-02-09

**Authors:** Anne‐Marie Lamhonwah, Ivo Barić, Jessica Lamhonwah, Marina Grubić, Ingrid Tein

**Affiliations:** ^1^ Division of Neurology Department of Pediatrics, and Genetics and Genome Biology Program The Research Institute The Hospital for Sick Children University of Toronto Toronto Ontario M5G 1X8 Canada; ^2^ Department of Pediatrics University Hospital Center Zagreb Zagreb 10000 Croatia; ^3^ School of Medicine University of Zagreb Zagreb 10000 Croatia; ^4^ Department of Laboratory Medicine and Pathobiology University of Toronto Toronto Ontario M5G 1X8 Canada

**Keywords:** Attention deficit/hyperactivity disorder, cardiomyopathy, carnitine‐responsive, myopathy, OCTN2 deficiency

## Abstract

This boy presented with ADHD at 3 years and at 8 years was hyperactive with no documented hypoglycemia and had myopathy, cardiomyopathy, and very low serum carnitine. L‐carnitine improved his exercise intolerance, cardiomyopathy, and behavior. Analysis of *SLC22A5* revealed a premature stop codon (p.R282*) and a novel in‐frame deletion (p.T440‐Y449).

## Introduction

Primary systemic carnitine deficiency (OMIM 603377) is due to a defect in the plasmalemmal high‐affinity carnitine/organic cation transporter (OCTN2) which is ubiquitously expressed 1. This is a potentially lethal, autosomal recessive disorder characterized by early childhood‐onset carnitine‐responsive cardiomyopathy, myopathy, recurrent hypoglycemic hypoketotic encephalopathy, failure to thrive and extremely low plasma and tissue carnitine concentrations (<5% controls) 1. There is microvesicular steatosis in muscle and liver and impaired renal tubular reabsorption of carnitine. Early diagnosis and lifelong treatment with high‐dose oral L‐carnitine supplementation is lifesaving and reverses the pathology including the cardiac, motor, and hepatic dysfunction with cessation of episodic coma and a restoration of normal growth, although serum and tissue concentrations remain low due to the persisting renal leak of carnitine arising from the OCTN2 transporter defect.

In previous work, we have demonstrated strong expression of Octn2 in the olfactory nerve and bulb, molecular layer and neuronal processes of input fibers extending vertically in motor cortex, dendritic arborization of the cornu ammonis and dentate gyrus of the hippocampus, neuronal processes in the arcuate nucleus of the hypothalamus, choroid plexus and Purkinje cells of the cerebellum in murine brain 2. This expression pattern may suggest roles of Octn2 in the modulation of cerebral bioenergetics through maintenance of homeostasis in the critical acyl‐CoA/CoA pool and in acetylcholine generation for synaptic neurotransmission in olfactory, satiety, limbic, cognitive, memory, and motor functions through its transport of key cerebral substrates such as L‐carnitine, acetyl‐L‐carnitine, and other acylcarnitine esters. This distribution may play a role in the pattern of neurological injury that occurs in OCTN2 deficiency during catabolic episodes of hypoglycemic, hypoketotic coma, and/or may influence ongoing normal cerebral function, growth and development affecting the neurological phenotype. This case with OCTN2 deficiency highlights an associated ADHD in the absence of overt hypoglycemia in which L‐carnitine therapy may have contributed to the improvement of ADHD‐related symptoms.

## Case Study

All studies and procedures were performed with written informed consent and with approval and in accordance with the ethical standards of the Research Ethics Board of the Hospital for Sick Children, Toronto, ON, Canada and with the 1975 Declaration of Helsinki, as revised in 2000. Informed consent to publish this case was obtained from the parents of the proband.

This 8‐year‐old Croatian boy was admitted for investigation of elevated aminotransferases (threefold elevation) in the context of his sister's sudden death. There was no parental consanguinity. His sister was born following an uneventful pregnancy and delivery. She failed to thrive since 3 months of age. At 4.5 months, she suffered an upper respiratory tract infection with recurrent vomiting, pallor, hypotonia, and somnolence and suffered a cardiac arrest and died. Autopsy revealed a fatty liver. Her archived newborn screening Guthrie card revealed an extremely low total carnitine of 2.79 *μ*mol/L, free carnitine of 2 *μ*mol/L, acylcarnitines of 0.79 *μ*mol/L, and low long‐chain acylcarnitines consistent with a carnitine transporter (OCTN2) disorder.

The proband was born by Cesarian section at 38‐week gestation due to maternal hypertension. In infancy he underwent two procedures for vesicoureteral reflux with secondary hydronephrosis and at 6 years an inguinal hernia repair. He was diagnosed to have a significant attention deficit/hyperactivity disorder (ADHD) beginning at age 3 years. He was treated with piperidine for 3 months without success, and this was discontinued 1 week prior to his admission at 8 years and therefore no longer present given the short half‐life.

On admission at 8 years of age, he was pale and notably hyperactive with a short attention span. Weight was on the 10th centile. He was kyphotic with myopathy and mild muscle atrophy. Liver was palpable by 1 cm. Patellar reflexes could not be elicited; however, he had clonus of the left foot.

During formal psychological evaluation this boy was extremely hyperactive, easily distractible and impulsive with a shortened attention span both in educational and play activities compared with age‐matched peers. He could not remain still but frequently fidgeted, often standing up from his chair and interrupting others while speaking. Based upon parental reporting, these difficulties were persistent in the clinical, home, and school settings. Parents further reported that he was unable to recognize dangerous situations, and that he had difficulties in his interactions with other children, often disrupting their games, not waiting for his turn and disobeying rules, which made his behavior very difficult to control. On cognitive evaluation (WISC‐R), his full‐scale IQ (80) was within the low average range and he was diagnosed to have learning disabilities including dyslexia, dysgraphia, and problems with visuo‐spatial organization. On the Parent Report ADHD‐T test 3, his score was 140 consistent with severe ADHD (>99% of the population for hyperactivity, impulsivity, and attentional deficit subscales). He thereby fulfilled the DSM‐IV criteria for attention deficit/hyperactivity disorder.

On biochemical evaluation, his serum total carnitine was markedly reduced at 1.4 *μ*mol/L with a free carnitine of 1 *μ*mol/L and low long‐chain acylcarnitines. Renal tubular reabsorption of carnitine was negligible. Electromyography demonstrated myopathic features. Echocardiography and ECG revealed left ventricular hypertrophy with poor contractility.

On clinical suspicion of OCTN2 deficiency, he was treated with high‐dose oral L‐carnitine (100 mg/kg/day) which led to significant improvements in his cardiomyopathy (left ventricular ejection fraction before treatment was 35–44% and increased to 70–73% seven months after treatment), exercise intolerance and a remarkable improvement in his behavior as well as weight gain. Within a few weeks after initiation of the L‐carnitine, his school functioning and overall behavior improved even though certain behavioral and learning problems persisted. Based upon parental and teacher reports, he demonstrated improved concentration in his school work with reduced interruption of teachers and students during class. He still required assistance with reading and writing tasks but demonstrated longer concentration periods. His educational assistant noted the most significant improvements in his hyperactivity and attention span which were felt not to be solely attributable to behavioral therapy or maturation. His most persistent behavioral problems were related to disobedient behavior at home. As with all individuals with OCTN2 deficiency, he is lifetime dependent upon daily high‐dose carnitine supplementation 4.

He continued to suffer from ongoing behavioral and social adjustment problems in school, but was able to complete primary and vocational secondary school. Medication selection for the treatment of his ADHD was limited due to potential cardiac complications. As such, he underwent a trial of atomoxetine with limited success. At 20 years of age, he was sent to a supervised community setting for 18 months to improve his work habits. Afterward, he left this community and at the time of this article was working independently in gardening.

[^3^H]‐L‐carnitine uptake studies were performed in the proband's cultured skin fibroblasts by established methods in our laboratory 1. Molecular analysis of the *SLC22A5* gene was done on fibroblast genomic DNA from the proband and from the blood genomic DNA of his parents. The 10 exons were amplified by PCR. Nucleotide sequencing was done by the Sanger dideoxy method of the subclones of the amplicon 1. Immunoblot of the proband's fibroblasts was performed using our anti‐mOctn2 antibody 5.

## Results and Discussion

**Figure 1:**
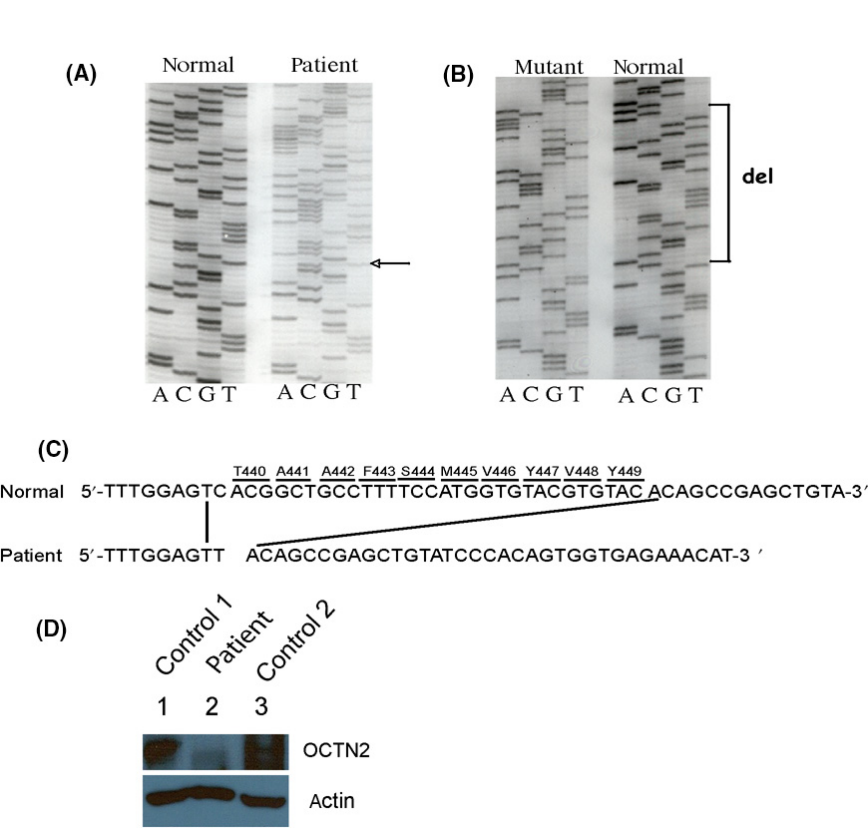
(A) Autoradiography of gel showing the sequencing data of exon 8 of the SLC22A5 gene encoding the organic cation/carnitine transporter OCTN2 in a normal control and the patient. The mixed sequences in patient (see arrow) indicate heterozygote alleles. (B) Nucleotide sequencing of subclones of PCR of exon 8 amplicon confirmed the presence of a mutant allele (deletion of 30 bases). (C) Deletion of the 30 bases indicates an in‐frame deletion of 10 amino acids (numbered T440‐Y449) in the OCTN2 protein. (D) Immunoblotting of fibroblast protein lysates from two control individuals (lanes 1 and 3) and patient (lane 2) with polyclonal anti‐mOctn2 antibody (upper panel) (band densities of Western blot as determined by ImageJ software are 32174, 12282, and 43921 for control 1, patient and control 2, respectively) and by monoclonal anti‐beta actin antibody (lower panel) (band densities of 5083, 5045, and 4188 for control 1, patient and control 2, respectively) (*n* = 2).

[^3^H]‐L‐carnitine uptake studies in cultured skin fibroblasts showed markedly reduced carnitine uptake (4% controls) consistent with our previous studies 4. Nucleotide sequencing of the fibroblast genomic DNA by the Sanger dideoxy method of the subclones of the amplicon spanning exons 7–9 of the *SLC22A5* gene revealed a novel, first time reported, in‐frame deletion of 30 nucleotides NM_003060.3 c.1582‐1611. There was maternal inheritance of this mutant allele which leads to an in‐frame deletion of 10 aa (p.T440‐Y449) (Fig. 1A–C). This 10 aa sequence is conserved 100% among all species sequenced to date (NCBI Blast). This deletion occurs in a putative caveolin‐1 binding site which may be important in posttranslational regulation of Octn2 trafficking to the cell surface which is dependent on formation of a multiprotein complex containing caveolin‐1 and regulated by PKC‐dependent phosphorylation as shown in rat astrocytes 6.

There was paternal inheritance of the allele harboring the c.844C>T (NM 003060.3) change in exon 5 (data not shown) that results in a premature stop codon p.R282* in the intracellular loop between transmembrane domains VI and VII in OCTN2 leading to a predicted truncated protein of 281 aa with loss of transmembrane domains VII to XII, likely resulting in a protein that is either rapidly degraded or not functional 1, 7. This nucleotide change was also detected by next‐generation sequencing using SOLiD methodology. The allelic frequency of this stop codon is 0.00004942 (exac.broadinstitute.org). Immunoblot of fibroblasts with anti‐mOctn2 antibody (which is a polyclonal rabbit antibody raised against the carboxy terminus of mOctn2 which specifically differentiates mOctn2 from mOctn1 and mOctn3) 5 revealed reduced expression of a truncated protein (Fig. 1D).

OCTN2 is expressed in many regions of the cerebral cortex including the arcuate nucleus of the hypothalamus (chemiosmotic appetite sensing system) and the cornu ammonis and dentate gyrus of the hippocampus (limbic and memory systems) 2. There is an extensive literature on the potential roles of L‐carnitine and acetyl‐L‐carnitine in the modulation of cerebral bioenergetics, neuroprotection, and acetylcholine generation for synaptic neurotransmission which may involve satiety, limbic, cognitive, and memory functions 2. There appears to be a competitive inhibition of L‐carnitine uptake by Octn2 in isolated rat cerebral cortex cells at very high concentrations of 1 mmol/L of GABA, which is a major inhibitory CNS neurotransmitter 8. The addition of GABA was also correlated with inhibition of carnitine acetyltransferase which is responsible for the delivery of acetyl moieties for acetylcholine synthesis, suggesting a negative feedback between acetylcholine and GABA 8. Acetyl‐L‐carnitine was also shown to counteract the age‐dependent reduction of several rodent CNS receptors such as nerve growth factor receptors, NMDA receptors in the hippocampus, frontal cortex and striatum and those of glucocorticoids and various neurotransmitters, thereby enhancing synaptic transmission 9. Acetyl‐L‐carnitine was further shown to have a stereospecific facilitatory action on neuronal responses to acetylcholine and 5‐hydroxytryptamine in single neurons of the rat medullary–pontine reticular formation 10. It has also been shown to have neuroprotective effects through modulation of neuronal energy processes and its antioxidant activities. Examples of its protective effects against amyloid‐beta peptide 1–42 include up‐regulation of heat shock proteins and cellular glutathione 11. Furthermore, it has been shown to up‐regulate the expression of a mitochondrial voltage‐dependent anion channel in rat brain which is important in calcium homeostasis and plays a role in apoptosis 12. Chronic acetyl‐L‐carnitine supplementation in healthy mice has been shown to increase monoamine neurotransmitters noradrenaline and serotonin content and to increase energy metabolism (adenosine nucleotides, phosphocreatine, and phosphocreatine/creatine ratio in the cortex) in murine brain 13. In a study of ^13^ C‐labeled acetyl‐L‐carnitine by ex vivo ^13^C‐NMR spectroscopy in immature rat forebrain, the acetyl moiety of acetyl‐L‐carnitine was metabolized for energy in both astrocytes and neurons and the label was incorporated into the neurotransmitters glutamate and GABA 14. In aged rats, acetyl‐L‐carnitine enhanced motor behavior in attention and exploratory activity 15. It also lowered brain lipid hydroperoxide levels, thereby improving learning and memory in senescence acceleration‐prone mice 16. In other studies, it has been shown to reduce impulsive behavior in adolescent rats by increasing noradrenaline levels in the cingulate cortex and the 5HIAA/5HT ratio in both medial frontal and cingulate cortex 17. Acetyl‐L‐carnitine has also been shown to prevent neuronal loss to and to improve memory function in parkinsonian rats by upregulating dopamine D1 receptors and attenuating microglial activation and release of inflammatory mediators 18.

L‐carnitine has similarly been shown to have neuroprotective and antioxidant roles. L‐carnitine was shown to modulate cortical electrical spike activity in cultured neuronal networks primarily through GABAA receptor activation 19. L‐carnitine has been shown to inhibit increases in glutamate, glycine, and superoxides and to decrease cardiolipin after hypoxia‐ischemia in newborn rats 20. In aged rats, carnitine was shown to act as a neuromodulator increasing dopamine, epinephrine, and serotonin levels 21. Thus, both L‐carnitine and acetyl‐L‐carnitine have been shown to have numerous roles in cerebral bioenergetics, neuroprotection, and neurotransmission which may thereby play pathophysiologic roles in cognition, behavior, and attention.

Previous studies of the use of L‐carnitine or acetyl‐L‐carnitine in attention deficit/hyperactivity disorder have yielded contradictory results. In one previous randomized, double‐blind, placebo‐controlled double‐crossover trial of the efficacy of carnitine in the treatment of boys with attention deficit/hyperactivity disorder, the investigators found a significant decrease in attention problems and aggressive behavior in 13/24 boys with ADHD with a decrease of 20–65% (8–48 points) in responsive boys as assessed by the Child Behavior Checklist (CBCL) total problem rating scale 22. In a placebo‐controlled trial, the use of acetyl‐L‐carnitine as an adjunctive therapy to methylphenidate in the treatment of ADHD in children and adolescents showed no differences with the placebo plus methylphenidate group 23. In another multi‐site, placebo‐controlled trial of acetyl‐L‐carnitine in 112 children with ADHD, the primary intent‐to‐treat analysis of 9 DSM‐IV teacher‐rated inattentive symptoms was not significant; however, secondary analyses revealed a significant moderation by subtype with superiority of acetyl‐L‐carnitine over placebo in the inattentive type, with an opposite tendency in combined type 24.

The association of a prominent attention deficit/hyperactivity disorder in the boy in our study may have been the result of cerebral injury arising from undetected episodes of hypoglycemia, although there was no history of overt episodes of hypoglycemia or perinatal injury raising the question of a primary role for L‐carnitine in the cerebral function, growth and development of our proband. Of note, children with medium‐chain acyl‐CoA dehydrogenase (MCAD) deficiency, which is the most common disorder of fatty acid oxidation, typically manifest with secondary carnitine deficiency, even in asymptomatic MCAD‐deficient children receiving normal dietary intake, in whom there is a low total plasma carnitine ranging from 10% to 50% of normal with a reduction in plasma‐free carnitine and a relative increase in the esterified fraction 25. In a follow‐up survey of 78 MCAD‐deficient survivors (>2 years of age), routine developmental assessment was abnormal in 29 patients (37%) and showed global developmental disability in 16 (21%), speech and language delay in 16 (21%), behavioral problems in 12 (15%), and attention deficit disorder in 9 (12%) 26, 27.

In our current case of OCTN2 deficiency, this boy's ADHD‐related symptoms appeared to improve following high‐dose L‐carnitine therapy. This case adds OCTN2 deficiency as a potential contributing factor to be considered in the differential diagnosis of a child with ADHD, in the case of very low serum carnitine concentrations, and underlines the importance of early identification and intervention with high‐dose oral L‐carnitine therapy which may not only reverse the cardiomyopathy, myopathy, and failure to thrive of OCTN2 deficiency, but may contribute to the improvement of the symptoms related to ADHD along with behavioral therapy and ADHD‐specific medications.

## Authorship

A‐ML: contributed to the design and conceptualization of the study, analysis, and interpretation of the data and to the writing and revision of the manuscript for intellectual content. IB: contributed to the design and conceptualization of the study, analysis, and interpretation of the data and to the writing and revision of the manuscript for intellectual content. JL: contributed to the analysis and interpretation of the data and to the writing and revision of the manuscript for intellectual content. MG: contributed to the analysis and interpretation of the data and to the writing and revision of the manuscript for intellectual content. IT: wrote the first draft of the paper and contributed to the design and conceptualization of the study, analysis, and interpretation of the data and to the revision of the manuscript for intellectual content.

## Conflict of Interest

Dr. Anne‐Marie Lamhonwah reports an operating grant from the Rare Diseases Foundation supporting in part this study. No personal fees were obtained. The Rare Diseases Foundation had no role in the study design, analysis of data or writing of the manuscript or decision to publish this manuscript. Dr. Ivo Barić declares that he has no conflict of interest. Ms. Jessica Lamhonwah declares that she has no conflict of interest. Dr. Marina Grubić declares that she has no conflict of interest. Dr. Tein reports an operating grant from the Rare Diseases Foundation supporting in part this study. No personal fees were obtained. Dr. Tein wrote the first draft. The Rare Diseases Foundation had no role in the study design, the collection, analysis, and interpretation of the data or the writing of the manuscript or decision to publish this manuscript. Dr. Tein also reports operating grants from the United Mitochondrial Diseases Foundation, The Physician's Services Incorporated Foundation of Ontario, the Foundation for Prader‐Willi Research, and the Myositis Foundation which did not support or have any influence on the current study.
